# Association between comorbid conditions and disease severity in COVID-19 patients: A retrospective study from a government medical college hospital of Central India

**DOI:** 10.6026/973206300223640

**Published:** 2026-06-30

**Authors:** Rakesh Kumar Sisodia, Tarunendra Kumar Mishra, Mahesh Kumar Patidar, Mahendra Chouhan, Umesh Sinha

**Affiliations:** 1 Department of General Medicine, Government Medical College Ratlam, Madhya Pradesh, India; 2 Department of Respiratory Medicine, Government Medical College Ratlam, Madhya Pradesh, India; 3 Department of Community Medicine, Government Medical College Ratlam, Madhya Pradesh, India

**Keywords:** Coronavirus disease 2019 (COVID-19), comorbidities, disease severity, hypertension, diabetes mellitus, severe acute respiratory syndrome coronavirus 2(SARS-CoV-2)

## Abstract

The COVID-19 pandemic has posed a major global health challenge, with emerging evidence showing worse outcomes among patients with pre- existing comorbidities. Therefore, it is of interest to examine the association between comorbidity status and disease severity among 384 laboratory-confirmed COVID-19 patients admitted to a government medical college in Central India. Patients were divided into comorbid(n=192) and non-comorbid (n=192) groups, with hypertension (69.27%), diabetes mellitus (36.98%) and cardiac disease (13.54%) being most prevalent. Fever (67.45%), cough (55.47%) and shortness of breath (36.20%) were predominant symptoms, while severe illness occurred in 29.69% of comorbid versus 11.46% of non-comorbid patients (x^2^=24.83, p<0.00001). Thus, we show that hypertension and diabetes markedly increase COVID-19 severity, emphasizing prioritized care and aggressive management of high-risk populations.

## Background:

The outbreak of severe acute respiratory syndrome coronavirus 2 (SARS-CoV-2) in December 2019 ushered in a global health crisis that transformed healthcare provision models in countries across the world in fundamental ways [1]. This led to the coronavirus disease 2019 (COVID-19), which quickly attained pandemic levels and impacted almost all countries, causing millions of deaths globally. Even though the disease has been extensively studied in terms of viral pathogenesis and treatment modalities, determining factors that predispose certain individuals to severe manifestations remains a challenging issue [2]. COVID-19 has demonstrated remarkable diversity in clinical manifestations, ranging from complete absence of symptoms to severe disease characterised by acute respiratory distress syndrome, multi-organ dysfunction and mortality [3]. This heterogeneity in disease presentation has stimulated extensive research into host factors that could influence the clinical course. Among the many determinants investigated, pre-existing comorbid conditions have consistently emerged as significant predictors of unfavourable outcomes [4]. The pathophysiology of SARS-CoV-2 infection involves viral entry via angiotensin- converting enzyme 2 (ACE-2) receptors that are highly concentrated on respiratory epithelial cells and other organ systems [5]. Subsequent viral replication triggers inflammatory cascades and immunological responses that in susceptible individuals may become dysregulated, resulting in cytokine storm syndrome and tissue damage. Patients with chronic conditions typically display altered immune function, baseline inflammatory states and diminished organ reserve that could impair their ability to mount appropriate antiviral responses [6]. International epidemiological data from the early pandemic period identified hypertension, diabetes mellitus, cardiovascular disease and chronic respiratory conditions as the most prevalent comorbidities among hospitalised COVID-19 patients [7]. Chinese studies demonstrated hypertension in approximately 15-30 per cent of hospitalised cases, while 7-20 per cent of severely affected patients had diabetes [8]. Subsequent studies from Western countries revealed even higher comorbidity rates, with large cohort studies in the United States reporting hypertension in more than half of hospitalised patients [9]. Mechanistic associations between specific comorbidities and COVID-19 severity have been extensively examined. Hypertension is linked to endothelial dysfunction, chronic vascular inflammation and alterations in ACE-2 expression patterns that could potentially facilitate viral entry and disease progression [10]. Diabetes mellitus affects both innate and adaptive immune responses through multiple mechanisms, including impaired phagocytic activity, cytokine dysfunction and glycation-induced tissue damage [11]. Cardiovascular disease provides minimal physiological reserve to sustain the haemodynamic stress of acute infection. India, bearing a substantial burden of non-communicable diseases, faced particular vulnerability during the pandemic. The prevalence of diabetes and hypertension among the Indian population has been progressively rising, with current estimates indicating that more than 200 million individuals are affected by these two conditions [12]. Nevertheless, despite experiencing one of the highest COVID-19 caseloads globally, detailed information defining the correlation between comorbidities and disease severity across Indian regions remains relatively limited [13]. Therefore, it is of interest to compare disease severity distribution between patients with and without comorbidities, while secondary objectives included characterising the comorbidity profile and clinical presentation patterns in this population.

## Materials and Methods:

## Study design and setting:

This retrospective record-based cross-sectional study was conducted at Government Medical College Hospital, Ratlam, Madhya Pradesh, India. The institution functioned as a designated COVID-19 treatment center during the pandemic and provided comprehensive care across the entire spectrum of disease severity, ranging from mild illness to critically ill patients requiring intensive care support.

## Study period and population:

The study included all laboratory-confirmed COVID-19 patients admitted to the hospital between January 2021 and March 2021. This period was selected to evaluate a relatively homogeneous cohort of patients during the pre-second-wave phase of the pandemic, thereby minimizing variations related to evolving viral strains and treatment protocols.

## Eligibility criteria:

## Inclusion criteria:

Patients were included if they fulfilled all of the following criteria:

[1] Laboratory-confirmed SARS-CoV-2 infection by reverse transcription-polymerase chain reaction (RT-PCR) or rapid antigen test (RAT).

[2] Age ≥18 years at the time of admission.

[3] Hospitalization at the study institution during the study period.

## Exclusion criteria:

Patients were excluded if they met any of the following criteria:

[1] Age below 18 years.

[2] Negative COVID-19 diagnostic test results.

[3] Pregnancy, due to distinct physiological and clinical characteristics that could influence disease presentation and outcomes.

## Data collection:

Data were retrospectively retrieved from the Medical Records Department using a standardized pre-designed data extraction proforma.

Information collected included:

[1] Socio-demographic characteristics (age, sex and area of residence).

[2] Clinical characteristics (presenting symptoms and duration of illness before hospitalization).

[3] Comorbidity profile (type and number of pre-existing chronic illnesses).

[4] Disease severity at admission.

Patient confidentiality was maintained throughout the data collection process and all records were anonymized prior to analysis.

## Operational definitions:

## Disease severity classification:

## Mild disease: 

Presence of upper respiratory tract symptoms without breathlessness or hypoxia.

## Moderate disease:

Respiratory rate of 24-29 breaths/minute and/or oxygen saturation (SpO_2_)of 90-94% on room air, requiring supplemental oxygen through a face mask or low-flow nasal cannula.

## Severe disease:

Respiratory rate ≥30 breaths/minute and/or oxygen saturation (SpO_2_)<90% on room air, requiring high-flow oxygen therapy, non-rebreather mask, or non-invasive ventilatory support.

## Comorbidity definitions:

[1] Hypertension: Documented blood pressure > 140/90 mmHg or current use of antihypertensive medication.

[2] Diabetes Mellitus: Fasting blood glucose ≥126 mg/dL, postprandial blood glucose ≥200 mg/dL, random blood glucose ≥200 mg/dL, glycated hemoglobin (HbA1c) ≥6.5%, or current antidiabetic treatment.

[3] Cardiac Disease: Documented coronary artery disease, valvular heart disease, or structural heart disease.

[4] Chronic Kidney Disease: Previously diagnosed chronic kidney disease under medical management or renal replacement therapy.

[5] Respiratory Disease: Chronic obstructive pulmonary disease, bronchial asthma, or previous history of pulmonary tuberculosis.

[6] Other Comorbidities: Thyroid disorders, malignancies, immunocompromised states and other chronic systemic illnesses documented in medical records.

## Sample Size Determination:

Sample size was calculated using the formula for comparing two proportions. Based on previous literature reporting severe COVID-19 disease in approximately 30% of patients with comorbidities and 15% of patients without comorbidities, assuming a 95% confidence level (α = 0.05), 80% power (β = 0.20) and a 1:1 allocation ratio, the minimum required sample size was estimated to be 170 patients per group (340 total participants). To compensate for incomplete records and improve statistical precision, the sample size was increased by 15%, resulting in a target of 196 patients per group. Consecutive sampling of all eligible admissions during the study period was undertaken. A total of 384 patients fulfilled the eligibility criteria and were included in the final analysis, comprising 192 patients with comorbidities and 192 patients without comorbidities after frequency matching by admission week.

## Statistical analysis:

Data were entered into Microsoft Excel and analyzed using Epi Info (Centers for Disease Control and Prevention, Atlanta, USA) and SPSS version 25.0 (IBM Corp., Armonk, NY, USA). Continuous variables were expressed as mean ± standard deviation (SD), while categorical variables were presented as frequencies and percentages. Comparisons between groups were performed using:

[1] Independent sample t-test for continuous variables.

[2] Pearson's chi-square test for categorical variables.

Odds ratios (ORs) with 95% confidence intervals (CIs) were calculated to determine the strength of associations between comorbidities and disease severity. A p-value <0.05 was considered statistically significant.

## Ethical considerations:

The study protocol was reviewed and approved by the Institutional Ethics Committee of Government Medical College, Ratlam. As the study was retrospective and utilized anonymized hospital records, a waiver of informed consent was granted. Confidentiality and privacy of patient information were strictly maintained throughout the study.

## Results:

A total of 384 COVID-19 positive patients meeting eligibility criteria were included in the final analysis. The demographic and clinical characteristics of the study population are presented in Table 1. Figure 1 (see PDF) shows that mild disease was more common among patients without comorbidities (87 cases), whereas severe disease was more frequent among patients with comorbidities (40 cases). This suggests that the presence of comorbid conditions is associated with increased disease severity. The mean age of the study population was 53.6 ± 17.2 years, ranging from 18 to 92 years. Males constituted the majority at 65.63% and urban residents represented 68.49% of admissions. The largest age group was above 60 years (34.90%), followed by 51-60 years (22.92%). Fever emerged as the predominant presenting symptom, affecting 259 patients (67.45%), followed by cough in 213 patients (55.47%) and shortness of breath in 139 patients (36.20%). Constitutional symptoms, including weakness (16.41%) and body ache (14.84%), were also commonly reported. The figure shows that hypertension (69.27%) was the most common comorbidity among patients, followed by diabetes mellitus (36.98%) and cardiac disease (13.54%). Other comorbidities such as thyroid disorders, bronchial asthma, COPD, malignancy, chronic kidney disease, and tuberculosis were observed less frequently. This indicates that cardiovascular and metabolic disorders constituted the major comorbid conditions in the study population (Figure 2 - see PDF). Among the 192 patients with comorbidities, the distribution of specific conditions is detailed in (Table 2). Hypertension was the most prevalent comorbidity, present in 69.27% of patients with underlying chronic conditions. Diabetes mellitus was the second most common condition at 36.98%, followed by cardiac disease at 13.54% (Figure 2 - see PDF). Among the 192 patients with comorbidities, analysis of concurrent conditions revealed that 118 patients (61.46%) had a single comorbidity, 54 patients (28.13%) had two comorbidities and 20 patients (10.42%) had three or more concurrent conditions (Table 3). Patients with comorbidities were significantly older, with a mean age of 61.2 ± 12.8 years compared to 45.9 ± 16.4 years in the non-comorbid group (p<0.001). Among comorbid patients, 51.04% were above 60 years of age, whereas only 18.75% of non-comorbid patients belonged to this age category. Male predominance was observed in both groups, though the proportion was higher among non-comorbid patients (69.79%) compared to comorbid patients (61.46%, p=0.038). Both groups demonstrated similar urban residence patterns (66.67% versus 70.31%, p=0.612) (Table 4). The primary outcome analysis revealed marked differences in disease severity distribution between comorbid and non-comorbid groups, as presented in (Table 5). Among patients with comorbidities, severe disease occurred in 29.69% compared to only 11.46% in those without comorbidities, representing approximately a 2.6-fold increase in severe disease risk. Conversely, mild disease was observed in 63.02% of non-comorbid patients versus 41.15% of comorbid patients. The chi-square analysis demonstrated a highly significant association between comorbidity status and disease severity (x^2^=24.83, p<0.00001) (Table 6). Patients with pre-existing comorbid conditions had 3.27 times higher odds (95% CI: 1.91-5.60) of developing severe disease compared to those without underlying chronic conditions. When moderate and severe categories were combined, comorbid patients demonstrated 2.44 times higher odds (95% CI: 1.62-3.68) of developing moderate-to-severe disease. Analysis by specific comorbidity type revealed that cardiac disease (46.15%), COPD (50.00%) and diabetes mellitus (36.62%) were associated with the highest proportions of severe disease. All major comorbidities demonstrated statistically significant associations with severe disease compared to patients without comorbidities (Table 7). Clinical presentations were similar between groups, with fever, cough and dyspnoea remaining the predominant symptoms in both. A trend toward higher dyspnoea prevalence was observed in the comorbid group (40.63% versus 31.77%, p=0.066), though this did not reach statistical significance. Chemosensory symptoms (anosmia and ageusia) were more frequently reported among non-comorbid patients (Table 8).

## Discussion:

The current study provides robust evidence of a significant association between pre-existing comorbidities and COVID-19 disease severity in a Central Indian population. The finding that 50% of all hospitalised patients had at least one comorbidity underscores the substantial burden of chronic diseases in this region and its considerable implications for pandemic management strategies. The demographic patterns observed in this study, characterised by male predominance (65.63%) and concentration in older age groups (mean age 53.6 years), align with trends consistently documented across diverse global populations [14]. The higher proportion of male patients likely reflects both biological susceptibility factors and behavioural elements. Sex-specific differences in ACE-2 receptor expression, immune response characteristics and hormonal influences havebeen proposed as potential contributors to the observed male predisposition to severe COVID-19 outcomes [15]. The concentration of cases among patients over 60 years of age (34.90%) supports the well-established relationship between advancing age and adverse COVID-19 prognosis. Age-related immunosenescence, combined with the increased prevalence of comorbid conditions in elderly populations, creates an exceptionally high-risk profile warranting enhanced clinical vigilance [16]. The symptom profile identified in this cohort, with fever (67.45%), cough (55.47%) and dyspnoea (36.20%) as predominant features, corresponds to establish COVID-19 clinical phenotypes documented internationally [17]. The relatively lower prevalence of chemosensory disturbances compared to certain Western populations may reflect genuine epidemiological differences related to viral strain characteristics or population-specific factors, though under-reporting in the context of retrospective data collection cannot be excluded. The comorbidity spectrum identified in this study, with hypertension (69.27%) and diabetes mellitus (36.98%) as the most prevalent conditions, mirrors findings from numerous international investigations. The high hypertension prevalence among comorbid patients is particularly notable and may reflect the substantial burden of cardiovascular risk factors in the Indian population [18]. Multiple pathophysiological mechanisms have been proposed to explain the heightened risk of severe COVID-19 in hypertensive patients, including chronic endothelial dysfunction, altered renin-angiotensin system activity and upregulated ACE-2 receptor expression facilitating viral entry. The finding that over one-third of comorbid patients had diabetes mellitus carries significant implications, given the well- documented effects of glycaemic status on immune function and infection susceptibility. Diabetic patients exhibit impaired neutrophil chemotaxis, diminished phagocytic capacity and cytokine dysregulation that compromise antiviral defence mechanisms [11]. Furthermore, chronic hyperglycaemia promotes tissue glycation and microvascular injury, potentially exacerbating COVID-19-associated organ damage. The primary finding of this study substantially higher disease severity among patients with comorbidities represents the most clinically significant outcome. Severe disease occurred in 29.69% of comorbid patients compared to 11.46% in non-comorbid patients, with odds ratio analysis confirming a 3.27-fold increased risk of severe disease (95% CI: 1.91-5.60, p<0.0001). This finding has profound implications for clinical practice and healthcare resource planning, consistent with systematic reviews and meta-analyses that have established the high-risk profile associated with various chronic conditions [2]. The pathophysiological basis for increased severity in comorbid patients is multifactorial. Chronic inflammatory states characteristic of hypertension and diabetes promote baseline endothelial activation and prothrombotic conditions that may synergise with COVID-19-induced vascular injury. Impairment of both innate and adaptive immune functions compromises viral clearance and increases susceptibility to bacterial superinfection. Pre-existing organ dysfunction from chronic disease reduces physiological reserve and capacity to tolerate acute illness-related haemodynamic stress [6]. Analysis by specific comorbidity type revealed that cardiac disease (46.15% severe), COPD (50.00% severe) and diabetes mellitus (36.62% severe) were associated with the highest proportions of severe disease. These findings emphasise the particular vulnerability of patients with cardiopulmonary comorbidities and underscore the importance of aggressive risk factor management in these populations. The presence of multiple comorbidities in 38.54% of patients with chronic conditions (28.13% with two comorbidities, 10.42% with three or more) highlights the complexity of managing these patients and the potential for synergistic risk amplification. The most common combination of hypertension and diabetes mellitus, observed in 25% of comorbid patients, represents a particularly high-risk phenotype warranting intensive monitoring and early therapeutic intervention. The significant age difference between comorbid patients (mean 61.2 years) and non-comorbid patients (mean 45.9 years) illustrates the challenge of separating independent effects of age and comorbidity on disease outcomes. These variables frequently co-occur and likely act synergistically to amplify risk. However, the highly significant association between comorbidity status and severity observed in this study (p<0.00001) suggests an independent contributory role of chronic conditions beyond age-related effects. The similar urban residence proportion in both groups (approximately 68-70%) likely reflects healthcare access patterns rather than genuine differences in disease incidence. Urban residents typically have greater awareness of COVID-19 symptoms, better access to testing facilities and fewer barriers to hospitalisation, potentially creating selection bias in hospital-based populations. These findings carry substantial implications for clinical practice and public health policy. Early identification of patients with comorbidities should trigger enhanced surveillance, consideration of early therapeutic interventions and appropriate triage to higher-acuity settings. Identifying these risk factors enables early risk stratification and prompt management of high-risk COVID-19 patients, potentially reducing morbidity and mortality [19]. Standardised risk stratification protocols incorporating comorbidity assessment may optimise resource allocation in capacity-constrained healthcare environments. From a preventive perspective, individuals with chronic conditions should receive priority consideration for vaccination programmes and targeted health education emphasising risk-reduction behaviours. Healthcare systems should develop specific protocols for managing high-risk patients, including lower thresholds for hospital admission, more intensive monitoring parameters and earlier initiation of available therapeutic interventions. Several limitations warrant acknowledgment. The retrospective design and reliance on medical records may have resulted in incomplete capture of comorbidities and symptoms, particularly conditions not directly relevant to acute COVID-19 management. The single-centre setting limits external validity to other populations and healthcare contexts. Mortality outcomes were not assessed, which would have provided additional prognostic information regarding the ultimate impact of comorbidities. The study period captured a specific phase of the pandemic that may not represent other periods characterised by different viral variants or therapeutic protocols. Despite these limitations, the study provides valuable region-specific data addressing important knowledge gaps regarding COVID-19 clinical characteristics in Central India. The adequate sample size (384 patients), comprehensive data collection and robust statistical analysis with multiple analytic approaches strengthen the validity of observed associations. The equal distribution between comorbid and non-comorbid groups achieved through frequency matching enhances the comparative analysis.

## Conclusion:

Pre-existing comorbidities represent a critical determinant of COVID-19 severity. This is shown by the significantly elevated risk among affected patients in Central India. Thus, we show the imperative for comorbidity-driven risk stratification to underpin clinical decision- making and resource allocation during respiratory pandemics. Integration of chronic disease profiling into standardized triage protocols will enhance outcomes for high-risk populations and strengthen future pandemic preparedness strategies.

## Figures and Tables

**Table 1:** Baseline characteristics of COVID-19 patients (N=384)

**Characteristic**	**Number (N=384)**	**Percentage**
**Gender**		
Male	252	65.63%
Female	132	34.37%
Area of Residence		
Urban	263	68.49%
Rural	121	31.51%
**Age Groups**		
18-20 years	8	2.08%
21-30 years	32	8.33%
31-40 years	54	14.06%
41-50 years	68	17.71%
51-60 years	88	22.92%
>60 years	134	34.90%
**Duration of Illness**		
≤3 days	184	47.92%
4-7 days	160	41.67%
>7 days	40	10.42%
**Disease Severity**		
Mild	200	52.08%
Moderate	105	27.34%
Severe	79	20.57%
Comorbidity Status		
Present	192	50.00%

**Table 2:** Distribution of comorbidities among COVID-19 patients(N=192)

**Comorbidity**	**Number of Patients**	**Percentage**
Hypertension	133	69.27%
Diabetes Mellitus	71	36.98%
Cardiac Disease	26	13.54%
Thyroid Disorders	16	8.33%
Bronchial Asthma	11	5.73%
COPD	6	3.13%
Malignancy	5	2.60%
Chronic Kidney Disease	4	2.08%
Tuberculosis	3	1.56%

**Table 3:** Distribution of multiple comorbidities(N=192)

**Number of Comorbidities**	**Number of Patients**	**Percentage**
Single comorbidity	118	61.46%
Two comorbidities	54	28.13%
Three or more	20	10.42%
The most common comorbidity combination was hypertension with
diabetes mellitus, observed in 48 patients
(25.00% of comorbid group).

**Table 4:** Comparative characteristics of comorbid and non-comorbid COVID-19 patients

**Characteristic**	**Comorbid (N=192)**	**Non-Comorbid (N=192)**	**p-value**
Age (Mean ±SD)	61.2 ±12.8years	45.9±16.4years	<0.001
Gender			0.038
Male	118(61.46%)	134(69.79%)
Female	74(38.54%)	58(30.21%)
Residence	0.612
Urban	128(66.67%)	135(70.31%)
Rural	64(33.33%)	57(29.69%)
Age Distribution			<0.001
18-20 years	1(0.52%)	7(3.65%)
21-30 years	4(2.08%)	28(14.58%)
31-40 years	10(5.21%)	44(22.92%)
41-50 years	26(13.54%)	42(21.88%)
51-60 years	53(27.60%)	35(18.23%)
>60 years	98(51.04%)	36(18.75%)
Duration of Illness			0.142
≤3 days	84(43.75%)	100(52.08%)
4-7 days	88(45.83%)	72(37.50%)>
>7 days	20(10.42%)	20(10.42%)

**Table 5:** Disease severity distribution by comorbidity status

**Disease Severity**	**Comorbid(N=192)**	**Non-Comorbid(N=192)**	**Total(N=384)**	**p-value**
Mild	79(41.15%)	121(63.02%)	200(52.08%)	<0.00001
Moderate	56(29.17%)	49 (25.52%)	105(27.34%)	
Severe	57(29.69%)	22(11.46%)	79(20.57%)	

**Table 6:** Risk of severe disease by comorbidity status

**Outcome**	**Comorbid(N=192)**	**Non-Comorbid(N=192)**	**Odds Ratio(95% CI)**	**p-value**
Severe Disease	57(29.69%)	22(11.46%)	3.27(1.91-5.60)	<0.0001
Moderate-Severe	113(58.85%)	71(36.98%)	2.44(1.62-3.68)	<0.0001

**Table 7:** Severe disease distribution by specific comorbidity type

**Comorbidity**	**Total Patients**	**SevereDisease**	**Percentage**	**p-value vs Non-comorbid**
Hypertension	133	42	31.58%	<0.0001
Diabetes Mellitus	71	26	36.62%	<0.0001
Cardiac Disease	26	12	46.15%	<0.0001
Thyroid Disorders	16	4	25.00%	0.089
Bronchial Asthma	11	4	36.36%	0.024
COPD	6	3	50.00%	0.012
No Comorbidity	192	22	11.46%	Reference

**Table 8:** clinical symptoms by comorbidity status

**Symptom**	**Comorbid (N=192)**	**Non-Comorbid(N=192)**	**p-value**
Fever	132(68.75%)	127(66.15%)	0.581
Cough	110(57.29%)	103(53.65%)	0.462
Shortness of Breath	78 (40.63%)	61 (31.77%)	0.066
Weakness	35(18.23%)	28(14.58%)	0.329
Body Ache	25(13.02%)	32(16.67%)	0.312
Sore Throat	7(3.65%)	10(5.21%)	0.452
Loss of Taste	4(2.08%)	9(4.69%)	0.158
Loss of Smell	1(0.52%)	5(2.60%)	0.099
